# A Comparative Analysis of Discrete Entropy Estimators for Large-Alphabet Problems

**DOI:** 10.3390/e26050369

**Published:** 2024-04-28

**Authors:** Assaf Pinchas, Irad Ben-Gal, Amichai Painsky

**Affiliations:** 1 School of Electrical Engineering, The Iby and Aladar Fleischman Faculty of Engineering, Tel Aviv University, Tel Aviv 6997801, Israel; 2 Industrial Engineering Department, The Iby and Aladar Fleischman Faculty of Engineering, Tel Aviv University, Tel Aviv 6997801, Israel; bengal@tauex.tau.ac.il (I.B.-G.); amichaip@tauex.tau.ac.il (A.P.)

**Keywords:** entropy estimation, high dimensions, discrete, uniform, deterministic, empirical distribution

## Abstract

This paper presents a comparative study of entropy estimation in a large-alphabet regime. A variety of entropy estimators have been proposed over the years, where each estimator is designed for a different setup with its own strengths and caveats. As a consequence, no estimator is known to be universally better than the others. This work addresses this gap by comparing twenty-one entropy estimators in the studied regime, starting with the simplest plug-in estimator and leading up to the most recent neural network-based and polynomial approximate estimators. Our findings show that the estimators’ performance highly depends on the underlying distribution. Specifically, we distinguish between three types of distributions, ranging from uniform to degenerate distributions. For each class of distribution, we recommend the most suitable estimator. Further, we propose a sample-dependent approach, which again considers three classes of distribution, and report the top-performing estimators in each class. This approach provides a data-dependent framework for choosing the desired estimator in practical setups.

## 1. Introduction

Entropy estimation has long been a central area of research, driven by its role as a metric for measuring the uncertainty of source information [1]. One persistent challenge is the estimation of entropy in scenarios involving a large alphabet and a small sample size. Such a scenario can occur, for example, in image recognition, where symbols represent RGB values. This setup is typically referred to as the large-alphabet regime, where the entropy estimators are shown to be biased [2] and the convergence rate can be slow [3].

Entropy estimation is used in a variety of fields, such as machine learning, cryptography, and data compression. Noteworthy applications include feature selection in machine learning [4] and the development and analysis of encryption methods in cryptography, particularly in the task of assessing entropy based on small sample sizes to obtain an estimator with minimal mean square error [5,6]. Additionally, in natural language processing, a compelling application arises in the form of word-sense induction, which is a technique used for word clustering [7]. For instance, the SemEval 2010 WSI task commonly exhibits a small average number of examples per word, while the count of sense clusters may be substantially higher, sometimes exceeding ten clusters per word in certain systems.

A variety of entropy estimators proposed in different research studies exhibit diverse performance in distinct scenarios [8]. Notably, in the large-alphabet regime, numerous studies have been conducted [9,10,11,12,13,14]. This research seeks to build upon these studies by analyzing the latest approach to entropy estimation using deep neural networks, with a specific emphasis on the large-alphabet regime, which poses challenges to conventional entropy estimation methods, such as those that rely on the *plug-in* principle [2,15,16]. The primary focus lies in entropy estimation using small samples drawn from multiple distributions, spanning from nearly deterministic to uniform.

This study extends the findings of a prior comparative analysis involving eighteen entropy estimators, as presented in [17], by incorporating two novel state-of-the-art estimators that are based on deep neural networks (DNNs) and polynomial approximation. The DNN-based estimator defined in [18] performs well in practice, while the *polynomial* estimator comes with many favorable performance guarantees [19]. Consequently, it focuses on a broader variety of large-alphabet regimes across a wide range of distributions. Finally, our study provides guidance for selecting the most favorable entropy estimator for different setups.

This paper is organized as follows: Section 2 outlines the preliminaries, including fundamental entropy definitions and tools. Section 3 delves into the various entropy estimators and associated comparison studies. Section 4 details the experimental settings in this study, while Section 5 presents the results of the analysis using a variety of statistical measures. Lastly, Section 6 concludes with insights gleaned from the analysis and potential directions for future research.

## 2. Preliminaries

Shannon entropy serves as an information-theoretic metric for evaluating uncertainty in a random variable. For a discrete random variable *X* with a given distribution $P=(p_{1},p_{2},...,p_{k})$ with an alphabet X of size |X|=k, Shannon entropy is defined as

(1) $$H\left(X\right)=-\sum_{x\in X}p_{x}log_{2}p_{x}.\;$$

Given a collection of *n* iid samples from *X*, denoted by $X^{n}={\left\{X_{i}\right\}}_{i=1}^{n}$, our goal is to estimate H(X) from the sample, $\hat{H}\left(X^{n}\right)$. The empirical distribution is defined by $\hat{P}=\left({\hat{p}}_{1},{\hat{p}}_{2},...,{\hat{p}}_{k}\right)$, where each sampled probability follows ${\hat{p}}_{x}=\sum_{i=1}^{n}1\left(X_{i}=x\right)/n$, where 1() is the indicator function.

To assess the accuracy of the studied estimators, we focus on the mean squared error (MSE) between the entropy and its empirical estimation, which is a popular measure for comparison, as noted in [17,18,19]. This measure includes the bias error and the variance error, making it an ideal candidate for measuring the entropy estimator’s quality. The MSE satisfies

(2) $$MSE\left(\hat{H}\right)=Var\left(\hat{H}\right)+B{\left(\hat{H}\right)}^{2},\;$$

where the bias of the estimator $\hat{H}\left(X\right)$ is defined as

(3) $$B\left(\hat{H}\right)=\hat{H}\left(X^{n}\right)-H\left(X\right).\;$$

For unbiased estimators, the MSE represents the variance of the estimator, while in the case of a small-variance estimator, as seen in distributions closer to uniform, the bias significantly affects the MSE calculation.

Although the MSE offers a reliable method for estimating the bias and variance of estimators, the use of root MSE (RMSE) presents an additional advantage. Namely, it amplifies the differentiation between the estimators, especially when the differences are small, and expresses the error in the same unit as the entropy (bits). Our analysis involves evaluating the RMSE of one hundred measurements of entropy estimation for each combination of entropy estimator, sample size, alphabet size, and distribution.

## 3. Entropy Estimators

### 3.1. Overview of Entropy Estimators

Over the years, a variety of entropy estimators have been introduced. This section presents a review of twenty-one entropy estimators recently introduced in various studies, while the explicit formulas of these entropy estimators are presented in Appendix A. The maximum likelihood estimator (*plug-in*) [20] is designed by using the entropy directly calculated from the empirical distribution

(4) $${\hat{H}}^{ML}\left(X^{n}\right)=-\sum_{x\in X}{\hat{p}}_{x}log_{2}{\hat{p}}_{x}.\;$$

It exhibits commendable results within the classical regime, typified by a large sample size and a small alphabet size. However, a significant negative bias is observed as the alphabet size increases [2]. In response to this bias, several estimators were developed, including the Miller–Madow correction (MM) [15], which corrects the bias by incorporating a constant dependent on the non-zero sample probability count. Additionally, the *jackknife* estimator [16] proposes a correction based on estimation using the *plug-in* on all samples, excluding the $j^{th}$ sample. These corrections provide notable improvements for slight deviations from the classical regime, but as the sample-to-alphabet size ratio (STA ratio) decreases, these methods exhibit a large bias. Building on these is the Best Upper Bound (BUB) estimator [2], which takes a more systematic approach by approximating the optimal polynomial to H(X) within the space of *n*-degree polynomials, where *n* is the number of samples. This space precisely corresponds to the class of estimators that, like the *plug-in*, are linear in histogram order statistics. This estimator demonstrates superior performance over previous *plug-in*-based estimators when dealing with a small sample size and a small STA ratio. Additionally, it is worth noting that all the estimators mentioned up to this point are non-Bayesian.

Another non-Bayesian alternative is the Grassberger entropy estimator (GSB) [10]. This estimator demonstrates improved computation time, as it closely resembles the *plug-in* estimator, with the distinction that the logarithms are substituted with a $G_{n}$ function of the form

(5) $$G_{n}=\psi \left(n\right)+{\left(-1\right)}^{n}\int_{0}^{1}\frac{t^{n-1}}{t+1}dt,\;$$

where ψ(·) is the digamma function, and the function is specified for integer values and can be precomputed through recursion. Although the GSB estimator is generally considered a reasonable trade-off between bias and variance, the Schürmann (SHU) [21] estimator has shown that enhancements, particularly in terms of bias reduction at the expense of increased variance, can be achieved by generalizing the $G_{n}$ function to a one-parameter family of functions, denoted by $G_{n}\left(\zeta \right)$.

The Chao–Shen estimator (CS) [22], also known as the coverage-adjusted estimator (CAE) [23], estimates the entropy by considering it as the summation of an unknown population $H\left(X\right)=\sum_{k}b_{k}$, where each element in this population is defined by $b_{k}=-p_{k}\log\left(p_{k}\right)$. Later on, it utilizes the Horvitz and Thompson estimator [24] to provide an estimation for the total population. Specifically designed for scenarios with small sample sizes, the CS estimator is also capable of handling dependent observations.

One more estimator in use is the James–Stein shrinkage (SHR) estimator [9]. This estimator adopts a unique strategy by averaging two dissimilar models: a high-dimensional model with low bias and high variance and a lower-dimensional model with a higher bias and lower variance. The regularization level is controlled by the relative weights assigned to these two models. To achieve this, a convex function is applied to the empirical distribution

(6) $${\hat{p}}_{x}^{SHR}=\hat{\lambda }t_{x}+\left(1-\hat{\lambda }\right){\hat{p}}_{x},\;$$

where $\hat{\lambda }\in \left[0,1\right]$ represents the shrinkage intensity, ranging from zero (no shrinkage) to one (full shrinkage), while $t_{k}$ denotes the shrinkage target, commonly defined as the probability of a uniform distribution. This estimator is designed to be effective in both the large-alphabet regime and the classical regime. Additionally, it can transform into one of the Bayesian estimators when there are variations in the parameters $t_{k}$ and $\hat{\lambda }$.

The Bonachela (BN) estimator, as introduced in [25], is designed for scenarios marked by a small sample size and an STA ratio greater than one. In such cases, there is typically a relatively small alphabet size, where the empirical probabilities are not negligible. The primary goal of this estimator is to simultaneously minimize bias and variance across a broad range of probabilities. This approach strikes a balance between minimizing bias and addressing variance, which is particularly crucial when analyzing small sample sizes characterized by significant statistical fluctuations. Notably, the BN estimator is recognized for its numerical simplicity in implementation.

Built upon the Good–Turing formula [26,27], the Zhang entropy estimator [12] focuses on recovering distributional characteristics within the subset of the alphabet not covered by the sample size *n*. This approach leads to a notable increase in estimation accuracy compared to the *plug-in* estimator for any distribution with finite entropy. Moreover, the proposed estimator exhibits bias decay that is exponential in *n*. In cases of an infinite alphabet, the rate of bias decay is influenced by the distribution’s tail behavior. An improvement for this estimator was given in [28].

The Chao–Wang–Jost estimator (CWJ) [14] takes a novel approach by reformulating Shannon entropy in terms of the expected discovery rates of new species relative to the sample size, represented by the successive slopes of the species accumulation curve. The estimator is derived by applying slope estimators obtained from an improved Good–Turing frequency formula [26]. In evaluations conducted on finite alphabet sizes with an STA ratio greater than 0.1, the CWJ estimator demonstrated superior performance compared to the CS, GSB, Zhang, and *jackknife* estimators [14].

Within Bayesian statistics, significant attention has been devoted to the selection of priors for entropy estimation. Jeffrey’s (JEF) prior [29], which is a symmetric Dirichlet distribution with the parameter $a_{x}=1/2$, has been demonstrated to asymptotically maximize Shannon’s mutual information between $X^{n}$ and *X* [30]. The Laplace (LAP) estimator [31], derived from the Bayes estimator of the Tsallis entropy under a uniform prior probability density ($a_{x}=1$), presents a modified perspective on the JEF estimator. The Schürmann–Grassberger (SG) estimator [32] extends the LAP estimator and numerically identifies that the most accurate estimates are achieved using a symmetric Dirichlet distribution with the parameter $a_{x}=1/k$ as the prior. Building upon these, the minimax prior (MIN) estimator [33] formulates the estimation problem as a risk function, aiming to minimize the guaranteed value of the estimate. In the case of solving for a multivariate hypergeometric distribution, the Bayes estimator with $a_{x}=\sqrt{n}/k$ as the prior is identified as the optimal solution. Additionally, the Nemenman–Schafee–Bialek (NSB) estimator [11] was also developed within the Bayesian framework, extending considerations to priors with a power-law dependence on probabilities, specifically within the Dirichlet family of priors.

The CDM estimator [34], short for centered Dirichlet mixture, serves as the prior for the Bayesian entropy estimator. It centers a Dirichlet distribution over all conceivable binary words around either an independent Bernoulli (DBer) or a synchrony (DSyn) distribution. Initially designed for estimating the entropy of neural spike trains, it has been extended to generalize to binary vector data. In comparison, the PYM estimator [35] is based on the Pitman–Yor mixture prior, implying a narrow prior distribution over *H*. Significantly, it has been demonstrated that this estimator remains consistent across a variety of distributions, particularly excelling in providing optimal estimations for distributions characterized by long-tail behavior.

The *polynomial* estimator [19] was developed through the approximation of entropy using the polynomial representation of variables in the form ϕ(x)=−xlogx. This method achieves a balance between the *plug-in* estimation and the polynomial approximation by evenly splitting the sample and incorporating observed frequencies in each subset. Additionally, the *unseen* estimator [13] was suggested through a linear programming-based approach that leverages the sample to characterize the “unseen” segment of the distribution. Without making a priori assumptions about the distribution, the identification of unseen domain elements becomes inherently uncertain. Nonetheless, there is an effort to estimate the “shape” or histogram of the unseen part of the distribution, essentially quantifying the occurrence of unseen domain elements within various probability ranges. With such a reconstruction, the entropy of the distribution, dependent solely on the shape/histogram, can be estimated. Both of these estimators were specifically designed for large-alphabet regimes with varying STA ratios.

Finally, the neural joint entropy estimator (NJ) [18] introduces an innovative solution by employing a neural network-based entropy estimator. It minimizes the cross-entropy loss and derives the estimated entropy by individually estimating each element of the sum of conditional entropies. This estimator is specifically tailored to address large-alphabet regimes and small STA ratios.

Besides the entropy estimators already mentioned, the literature also includes several other schemes, including KNN [36], KDE [37], B-Spline [38], and Edgeworth [39] estimators. However, many of these are not adequate for the large-alphabet regime; hence, they are not included in this study. Additionally, certain neural network-based estimators, primarily designed for estimating mutual information rather than directly measuring entropy, such as MINE [40], JS [41], and SMILE [42], are also excluded, as they fall outside the scope of this work.

### 3.2. Past Research on Comparison of Entropy Estimators

This section reviews earlier comparative research on entropy estimators in the large-alphabet regime, which is different from studies focusing on the classical regime [25]. It also differs from studies that introduce a new estimator and offer a brief comparison to others, such as in [12,13,14,18,19]. Starting with the analysis presented in [2], four estimators (*plug-in*, MM, BUB, and jackknife) were applied to both real and simulated neuron spike data. In the classical regime, convergence was observed among the *plug-in*, MM, and jackknife estimators as the sample size increased. However, as the settings changed to the large-alphabet regime, the bias increased and the BUB estimator consistently outperformed the others.

In [9], nine estimators (*plug-in*, MM, NSB, CS, SHR, MIN, SG, JEF, and LAP) were analyzed on a variety of Dirichlet and Zipf’s Law distributions. It was shown that NSB, CS, and SHR outperformed the rest, exhibiting comparable results. Furthermore, the paper explores the Bayesian-based entropy estimators and emphasizes the significance of selecting an appropriate prior distribution with the parameter $a_{x}$, asserting that an improper choice of prior can result in poorer performance than the *plug-in* estimator.

The study in [17] examined eighteen estimators, namely, MIN, LAP, SG, JEF, CS, *unseen*, *plug-in*, *jackknife*, NSB, BN, CDM, GSB, SHR, BUB, Zhang, CJ, MM, and SHU, by analyzing them on samples from a uniform distribution with large and small alphabet sizes. The findings revealed that the SHR estimator outperformed the others for samples from the large alphabet size. However, for samples from the small alphabet size, the use of both the MM and SHR estimators was suggested. Furthermore, the study demonstrated that the SHR estimator achieved the least bias and also attained the lowest MSE, which approached zero.

In summary, although research has been conducted on the comparison of entropy estimators in the large-alphabet regime, this study sets itself apart in several key aspects. First, this work focuses on two novel state-of-the-art estimators, namely, NJ (DNN-based) and *polynomial* estimators. Second, it examines a broader range of distributions, ranging from uniform to deterministic. Last, the study introduces novel conclusions for selecting favorable entropy estimators based on their empirical proximity to the uniform distribution, a concept not explored in prior research.

## 4. Experimental Methods and Materials

### 4.1. Experimental Settings

To ensure a comprehensive exploration of distributions ranging from uniform to deterministic, we examine different parametric distributions on a variety of parameter values. First, we examine the uniform distribution as a special case for estimating the limits of the estimators. Second, we examine the geometric distribution with the parameter p∈[0,1], covering a wide range of distributions. Last, we examine the Zipf’s Law distribution with the parameter α∈[0.001,3.4]. The upper limit is set to 3.4 since, above this value, the estimator’s performance on the distributions shows consistent results.

The experiments cover a wide range of sample and alphabet sizes, separated into two main scenarios. In the first scenario, the alphabet size remains constant at $k=10^{5}$, with a varying sample size ranging from 100 to $10^{4}$, similar to previous studies [12,13,18,19]. The second scenario, akin to prior studies [12,13,14], considers a varying alphabet size ranging from 100 to $10^{5}$ and a fixed sample size of n=1000. These scenarios, representative of the large-alphabet regime, are studied, and the performance of the entropy estimators is analyzed.

### 4.2. The Implementation of the Entropy Estimators

The *plug-in*, MM, and *polynomial* entropy estimators were implemented using the “entropy” Python package Version 1.0 [19]. The SG, MIN, CS, and SHR estimators were derived from the “Entropy” R package Version 1.3.1 [43]. The Zhang estimator was sourced from the “EntropyEstimation” R package Version 1.2 [44]. Additionally, the *jackknife*, GSB, and CWJ implementations are available in the “Entropart” R package Version 1.6.–13 [45]. The BUB estimator was adopted from [2], as was *unseen* [13]. CDM and PYM are accessible in [46,47], respectively. The NJ estimator is provided through [48], as is NSB [49].

## 5. Results

### 5.1. Varying Sample Size with Fixed Alphabet Size

The analysis of twenty-one entropy estimators using the previously specified experimental settings led to an initial conclusion that some estimators produce results that are either incompatible or redundant, reducing the total number of estimators to seventeen. The SHU estimator was excluded due to its similar behavior to GSB in the large-alphabet regime for the RMSE and bias analyses, thereby making its inclusion redundant for the comparison. The BN estimator was also eliminated, as it proved to be the least compatible compared to the other estimators in the analysis. Similarly, the Bayesian estimators, including JEF and LAP, were found to be unsuitable for the large-alphabet regime. These particular estimators exhibit a higher bias relative to the rest and result in a high RMSE when the distributions approach the deterministic end.

**Figure 1:**
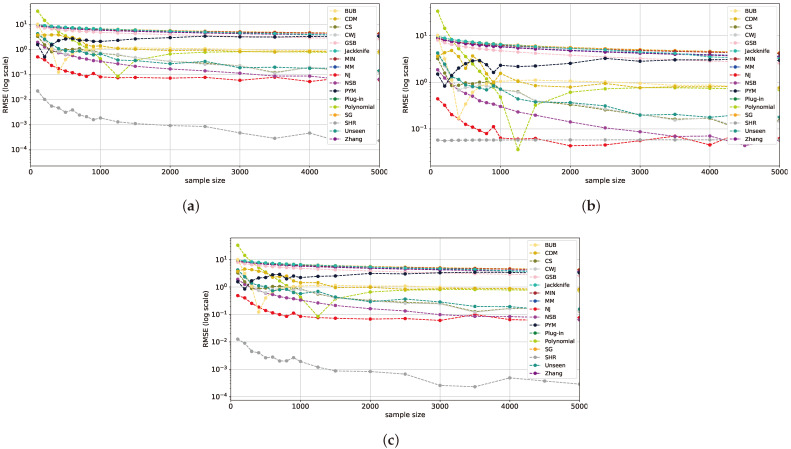
RMSE plots for multiple sample sizes and a large alphabet size of $10^{5}$ for a uniform distribution (**a**), geometric distribution with $p=10^{-5}$ (**b**), and Zipf’s Law distribution with α=0.001 (**c**). The y-axis representing the RMSE is on a log scale to better differentiate between the estimators.

We begin our analysis with uniform and near-uniform distributions, such as the geometric (with $p=10^{-5}$) and Zipf’s Law (with α=0.001) distributions, as shown in Figure 1. First, we observe that the SHR estimator outperforms all others in both the uniform and Zipf’s Law distributions, surpassing the NJ and NSB estimators, while in the geometric distribution, the NJ and SHR estimators achieve comparable results. The results of the SHR estimator align with the findings of [17] for a uniform distribution. This behavior also extends to near-uniform distributions. NJ and NSB exhibit similar behavior, with NJ outperforming NSB by nearly one order of magnitude at sample sizes below 1000. Furthermore, the sharp point-wise improvements in the RMSE of the *polynomial* estimator can be attributed to its estimation initially intersecting and then surpassing the actual entropy from below. As the sample size increases, this leads to a convergence to a value lower than the true entropy.

**Figure 2:**
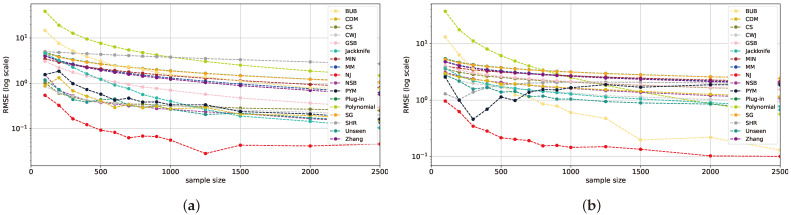
RMSE plots for multiple sample sizes and a large alphabet size of $10^{5}$ on a geometric distribution with p=0.001 (**a**) and Zipf’s Law distribution with α=1 (**b**).

Continuing the analysis of distributions deviating from the uniform distribution, referred to as the mid-uniform range, the NJ estimator consistently outperforms all others, often by an order of magnitude, while the NSB, *unseen*, *jackknife*, and BUB estimators present comparable results to the second best, as seen in Figure 2. This pattern continues in geometric distributions with *p* varying from 0.0001 to 0.01 and in Zipf’s Law distributions where α varies from 0.4 to 1.4. This continues until the point where NJ loses its superiority and attains results similar to those of other estimators across various sample sizes, as will be further discussed in Section 5.4. This excellent performance is attributed to NJ’s ability to generalize well, especially for small sample sizes [18].

**Figure 3:**
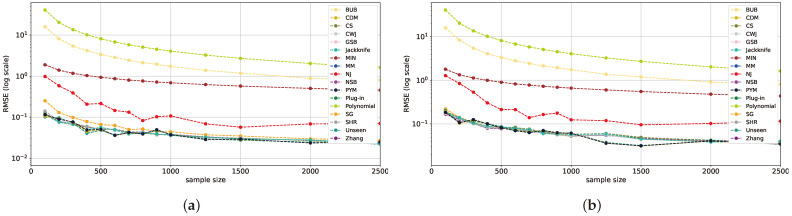
RMSE plots for multiple sample sizes and a large alphabet size of $10^{5}$ on a geometric distribution with p=0.9 (**a**) and Zipf’s Law distribution with α=3 (**b**).

At the deterministic end of the distribution spectrum (Figure 3), the classical estimators and their modifications, such as Zhang, PYM, MM, SG, SHR, NSB, GSB, *unseen*, *jackknife*, CWJ, *plug-in*, CS, and CDM, converge to one another, outperforming all others across varying sample sizes. This range is defined by an effectively smaller alphabet size, given the low probability of the majority of values—a characteristic of long-tail distributions, resembling the classical regime.

Notice that the *polynomial*, BUB, MIN, and NJ estimators are also not competitive within this range, suggesting that these estimators may not be well matched for deterministic-like distributions.

In conclusion, a summary of all the best-performing estimators for each distribution range is presented in Table 1. It can be seen that in the near-uniform distribution range, SHR, NJ, and NSB exhibit notable performance, where the SHR estimator outshines the other two. As the distributions deviate from uniform in the mid-uniform range, NJ emerges as a preferable choice. In the near-deterministic range, classical estimators based on the *plug-in* gradually converge, as shown in [17]. This range is characterized by a relatively small alphabet size compared to the sample size, resembling the classical regime.

### 5.2. Varying Alphabet Size with Fixed Sample Size

**Figure 4:**
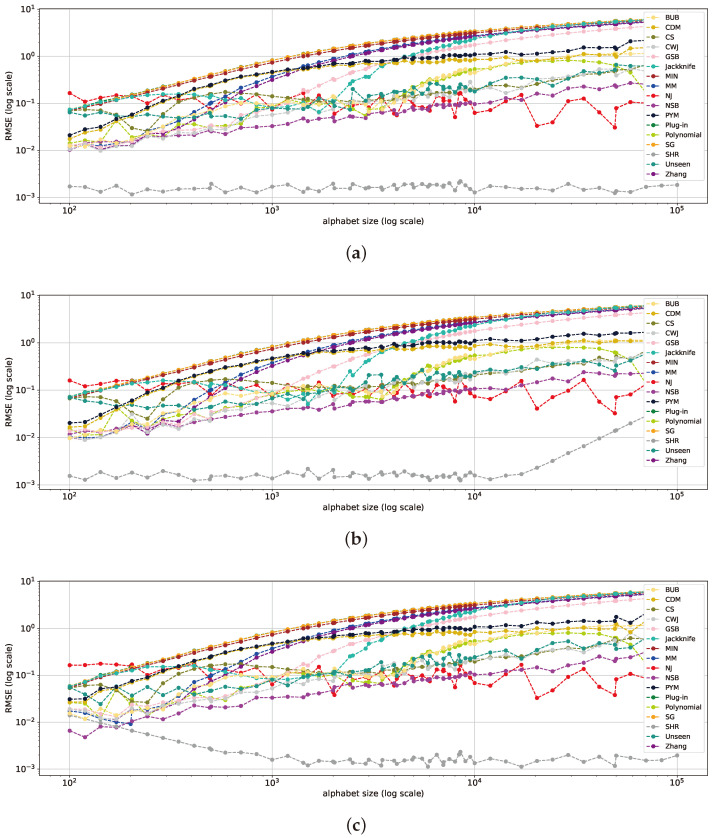
RMSE plots for multiple alphabet sizes with a constant sample size of 1000 for a uniform distribution (**a**), geometric distribution with p=0.00001 (**b**), and Zipf’s Law distribution with α=0.001 (**c**).

In this setup, a variety of alphabet sizes are analyzed, all maintaining a constant sample size of n=1000. Beginning with the uniform distribution, as depicted in Figure 4a, the SHR estimator emerges as the superior scheme, outstripping all other estimators by nearly an order of magnitude. Next, we observe that the NSB estimator exhibits the second-best results. The estimators’ performance is relatively stable across varying alphabet sizes. Notice that as the alphabet size increases, there is an increase in the RMSE of the estimators. This pattern is also reflected in the nearly uniform geometric distribution with $p=10^{-5}$ in Figure 4b and Zipf’s Law distribution with α=0.001, as depicted in Figure 4c.

The superior performance of the NSB, SHR, and NJ estimators is reflected across varying alphabet sizes. These results are in line with the findings of [9] regarding the excellent performance of the NSB and SHR estimators and the conclusions in [18] about NJ’s robust generalization abilities in the large-alphabet regime. For the remaining estimators, the bias increases with the alphabet size, observable in GSB, *plug-in*, MM, *jackknife*, Zhang, SG, and MIN. These estimators also converge from a certain alphabet size. The performance of *plug-in*, MM, and *jackknife* is consistent with the findings in [2] within the large-alphabet regime.

**Figure 5:**
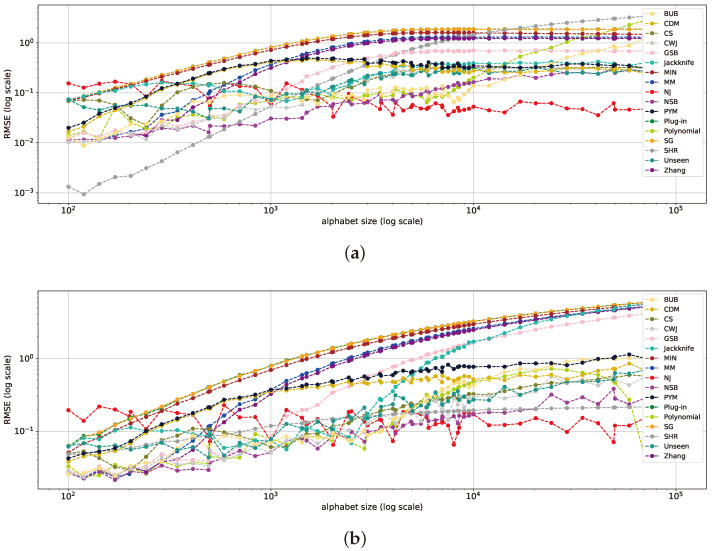
RMSE plots for multiple alphabet sizes with a constant sample size of 1000 on a geometric distribution with p=0.001 (**a**) and Zipf’s Law distribution with α=0.4 (**b**).

As the distributions deviate from uniform in the mid-uniform range, the performance of NJ and NSB remains consistently superior across the alphabet range, as seen in Figure 5. In addition, the PYM, CDM, *jackknife*, and *unseen* estimators also show comparable patterns and attain the second-highest results as the size of the alphabet increases. However, in specific ranges, the SHR estimator shows superior performance. The SHR takes the lead particularly when dealing with smaller alphabet sizes, aligned with the findings of [17].

**Figure 6:**
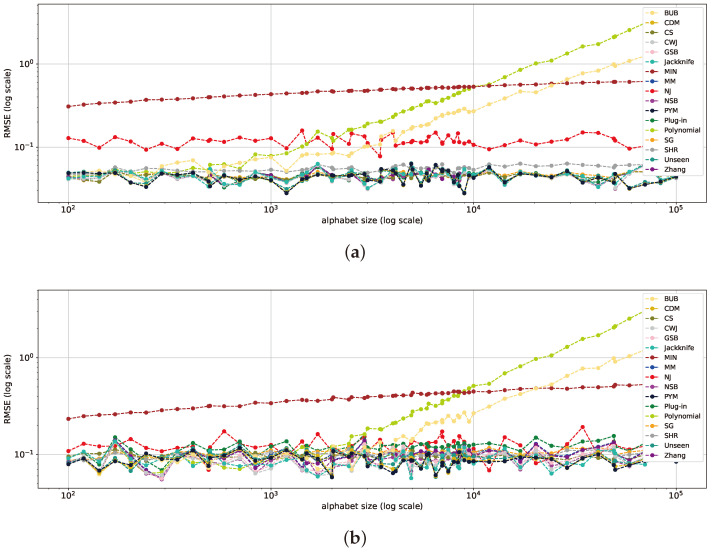
RMSE plots for multiple alphabet sizes with a constant sample size of 1000 on a geometric distribution with p=0.5 (**a**) and Zipf’s Law distribution with α=2 (**b**).

In the near-deterministic range (Figure 6), as was shown in the previous section, convergence is exhibited by the majority of the estimators, including *plug-in*, MM, NSB, Zhang, CWJ, GSB, SG, *unseen*, CDM, and PYM, demonstrating similar patterns across the alphabet range. This convergence is largely consistent, resulting in an RMSE of less than 0.1 across the full alphabet range, and aligns with previous findings [2,35]. However, as the alphabet size increases, the MIN shows a decline in performance, which can be attributed to its assumed prior, which depends more on the alphabet size than the sample values, resulting in an estimated probability similar to the uniform distribution in the large-alphabet regime. In the same vein, the RMSEs of the *polynomial* and BUB estimators increase when the alphabet size crosses the 1000 mark due to the former’s lack of correct polynomial coefficients and the latter’s bias.

To conclude, the most favorable estimators in each distribution range are similar to the ones obtained in Section 5.1 and presented in Table 1. This reveals that the SHR estimator significantly surpasses others in the near-uniform range, maintaining an almost steady RMSE of 0.001. In the mid-range, NJ shines with larger alphabet sizes, while SHR continues to lead for smaller ones. At the far-uniform end, the *plug-in* and other estimators designed for the classical regime converge and deliver top performance. The analysis of varying alphabet sizes strengthens the findings from Section 5.1, emphasizing that the performance of each estimator is more reliant on the distribution range than the alphabet size. An additional interesting conclusion is that the NJ estimator shows a steady performance across all experiments with varying alphabet sizes. This suggests that it is quite robust to the alphabet size.

### 5.3. Bias Analysis

**Figure 7:**
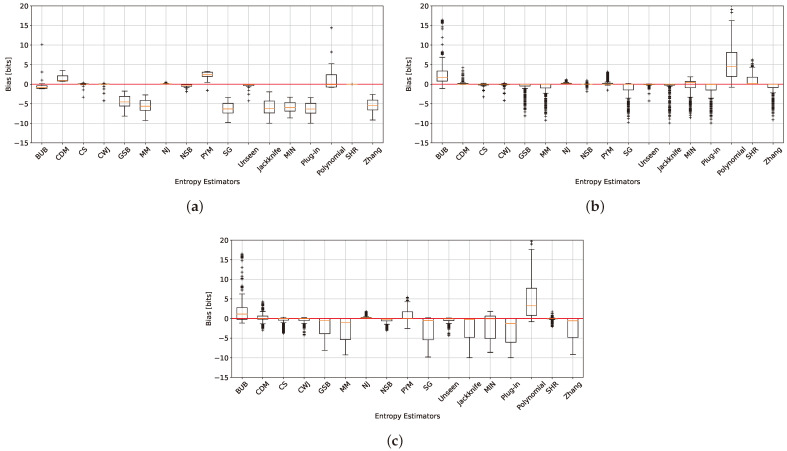
The bias in bits across all entropy estimators for uniform (**a**), geometric (**b**), and Zipf’s Law (**c**) distributions with an alphabet size of $10^{5}$ and varying sample sizes.

Let us now study the bias term of the entropy estimators. From (3), it can be inferred that overestimating the actual entropy leads to a positive bias, while underestimating it results in a negative bias. As demonstrated in [2], the *plug-in* estimator is negatively biased in both the large-alphabet and classical regimes. For uniform distributions (Figure 7a), the NJ and SHR estimators demonstrate the least bias, nearly approaching zero, a result consistent with the findings in Section 5.1 and Section 5.2. Geometric distributions are depicted in Figure 7b. The CDM, PYM, CS, CWJ, NJ, NSB, and *unseen* estimators deliver the best outcomes, with the NJ estimator outperforming all others. This aligns with NJ’s performance in [18] and the design of PYM to tackle long-tailed distributions [35], as well as the findings in Section 5.1 in the mid- and far-uniform distribution range.

In the case of the Zipf’s Law distribution shown in Figure 7c, the CS, CWJ, NJ, NSB, *unseen*, and SHR estimators perform the best, with NJ showing only a slight positive bias. This mirrors the geometric distribution bias analysis, except that the SHR estimator performed significantly better, as it is more suited for Zipf’s Law distributions, as outlined in [9].

In total, the NJ, NSB, CS, *unseen*, CWJ, CDM, and SHR estimators outperform all others, with NJ standing out as the superior one (Figure 7). Notably, PYM’s performance on the geometric distribution also exhibits the minimum bias. This result is not surprising, as the method is tailored for distributions with long exponential or power-law tails [35]. The largest bias is introduced by the *polynomial* estimator due to an inadequate polynomial fit. The remaining estimators exhibit a moderate bias size, which can vary depending on the setting.

### 5.4. Distributions According to Parameter Analysis

**Figure 8:**
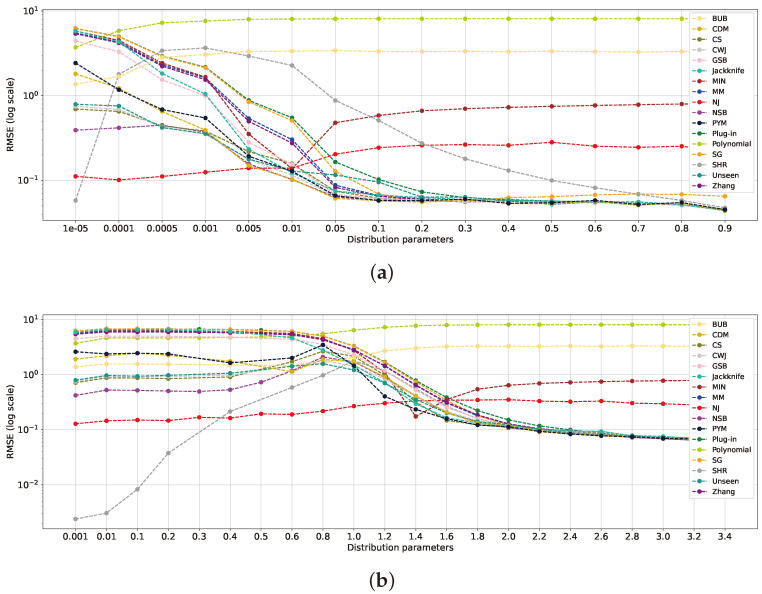
Geometric and Zipf’s Law distributions’ mean RMSEs of estimators at varying sample sizes and a fixed alphabet size of $10^{5}$ for each respective distribution parameter, with geometric *p* in (**a**) and Zipf’s Law α in (**b**).

We evaluate the performance of entropy estimators across the parameters of parametric distributions using the settings defined in Section 5.1. By examining the mean RMSE for each distribution, it can be seen that for each distribution family, distinct regions emerge where some estimators perform better than others. Specifically, within the geometric distributions, as depicted in Figure 8a, the SHR estimator excels over the others when $p\leq 10^{-5}$, in line with the findings in Section 5.1. This distribution, falling into the near-uniform range, mirrors results obtained for the uniform distribution in the previous section. For *p* values ranging from 0.0001 to 0.005, the NJ method outperforms all others, with NSB and *unseen* ranking as second best, as discussed in prior sections. When *p* exceeds 0.005, the distributions become more deterministic, resembling the classical regime. Here, NSB, CDM and CWJ converge and yield the best overall results within the near-deterministic range.

Continuing with the analysis of the Zipf’s Law distribution family (Figure 8b), the SHR estimator stands out as the top performer when α ranges from 0.001 and 0.4, a finding consistent with the results in the previous sections in the near-uniform range. For α values between 0.4 and 1.4, the NJ estimator outperforms the rest, obtaining a slightly higher RMSE than the top three estimators in the previous range. This also corresponds to NJ’s well-documented ability to generalize effectively [18]. Intriguingly, SHR performs second best, as the distribution remains closer to the uniform distribution. However, beyond a certain point, PYM outperforms the others, as it is better adapted to long-tail exponential distributions [35]. As α reaches 1.4 and beyond, the classical estimators converge, with the best performers listed in Table 1 in the near-deterministic range.

Overall, the analysis of the two distribution families reveals similar results, which are consistent with the findings in previous sections. Each type of distribution can be divided into three primary ranges: the near-uniform, mid-uniform, and far-uniform ranges. Within each range, the top-performing estimators tend to be similar, as described in Table 1.

### 5.5. Analysis of Total Variation Distance from Uniform Distribution

We now propose a different analysis that does not depend on the (unknown) underlying distribution *p*. Here, we consider the total variation (TV) between $\hat{P}$ and a uniform distribution. Formally, we define the *empirical total variation* (ETV) as

(7) $$ETV\left(P_{uniform},\hat{P}\right)=\frac{1}{2}\sum_{x\in X}\left|\frac{1}{k}-{\hat{p}}_{x}\right|.\;$$

This measure only depends on the sample and the alphabet size *k*. It can be evaluated in practice and can hence help one choose the preferred estimator in practical setups.

**Figure 9:**
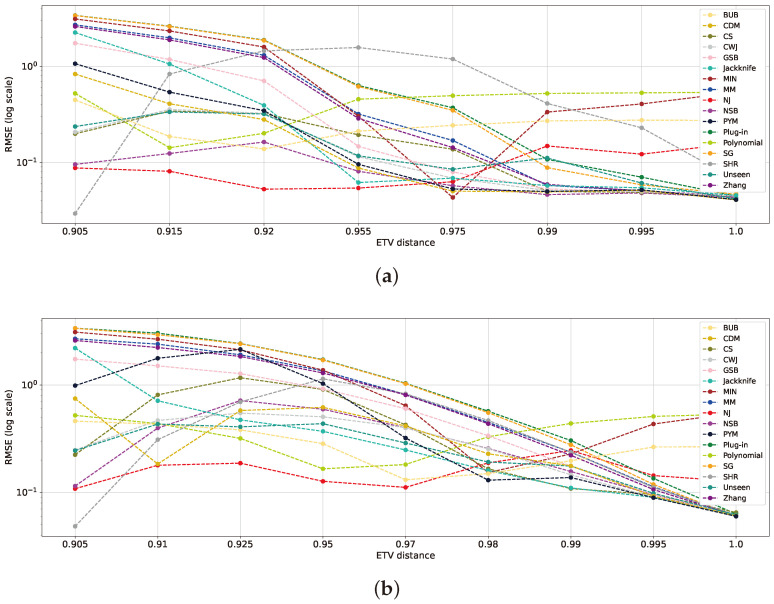
RMSE plots of estimators for the geometric and Zipf’s Law distributions with a sample size of n=1000 and an alphabet size of $k=10^{4}$. For each drawn sample, we evaluated its ETV and computed its corresponding mean RMSE for different entropy estimators. The geometric distributions are presented in (**a**), while the Zipf’s Law distributions are in (**b**).

Notice that in a large-alphabet regime, n≤k, the minimum ETV is obtained for uniform and empirical uniform distributions, $ETV(P_{uniform},\hat{P})=1-n/k$. On the other hand, its maximum value is achieved for a degenerate distribution, which leads to $ETV(P_{uniform},\hat{P})=1-1/k$. In our study, we set a sample size of n=1000 and an alphabet size of $k=10^{4}$, which leads to ETV∈[0.9,1]. Figure 9 illustrates the ETV for the Zipf’s Law and geometric distributions in the specified scenarios. The mean RMSE is evaluated at every distance for each estimator. As demonstrated in Figure 9a, the geometric distributions reveal three ranges for low ETV, mid-ETV, and high ETV. The low ETV falls below 0.91, the mid-ETV spans from 0.91 to 0.97, and the high ETV extends from 0.97 to the maximum distance of 1. Notably, NSB, SHR, and NJ yield the best results for low ETV values, while NJ excels in the mid-ETV range. The classical estimators surpass the others in the high-ETV range. These findings align with those noted in previous sections while also providing insight into their specific behaviors. Figure 9b presents the three ranges found in Zipf’s Law distributions with similar boundaries.

Another observation is that certain estimators, such as the *polynomial*, BUB, MIN, and NJ, exhibit increasing errors as the distance grows. These estimators do not perform competitively in the classical regime, as the distribution tends toward a deterministic end, and the classical estimators tend to yield better results. These insights are consistent with the previous analyses in Section 5.1 and Section 5.2. The SHR estimator shows distinct behavior, initially presenting the best outcome within the low-ETV range, as shown in previous studies [17]. However, it is soon overtaken by other estimators as the distribution distance for the uniform distribution increases, as its design better suits the low- and high-ETV ranges. Finally, the sharp improvement in the MIN estimator can be attributed to its prior matching the geometric distribution within this range. This reaffirms the claim made in previous works that claim that the selection of the prior significantly impacts the performance of Bayesian estimators [9].

### 5.6. Choosing the Most Favorable Entropy Estimator

Based on the analysis above, we can draw the following conclusions. The top estimators for each range are ranked based on their performance, with the first one being the most favorable. If the ETV distance falls in the low-ETV range, NJ, SHR, NSB, and CS are the most suitable. It is worth mentioning that the three estimators NJ, NSB, and SHR present competitive estimations due to their ability to handle distributions similar to a uniform one, as outlined in the preceding sections.

For an ETV distance within the mid-ETV range, the recommendation is to use the NJ estimator, followed by BUB, *unseen*, CDM, and NSB. NJ stands out as the overall best in this range, a finding that is consistent with previous sections and its capacity to generalize in the large-alphabet regime [18]. NSB, with its mixed Dirichlet priors, also demonstrates strong performance due to its ability to capture various distributions with its broadly defined prior [11].

Finally, for high ETV values, the traditional estimators converge and produce the best results. The top performers are the PYM, *jackknife*, *unseen*, CDM, and NSB estimators. These results align with previous sections.

**Figure 10:**
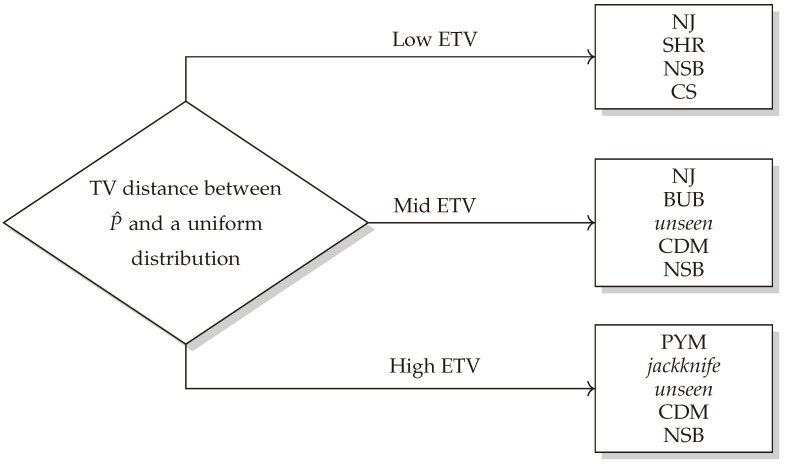
A decision tree for selecting the most effective entropy estimator for an unknown distribution in the large-alphabet regime.

In summary, the proposed selection method based on the analysis presented in the previous sections is specifically engineered to achieve the most favorable entropy estimation in the large-alphabet regime (Figure 10). Interestingly, the NSB estimator consistently performs well across all ranges and can be a good starting point when estimating an unknown distribution. Despite NJ outperforming the other two estimators in the low- and mid-ETV ranges, its long computation time lessens its appeal as a first choice. It is important to emphasize that the ETV cut-off ranges depend on the choice of *n* and *k*. Thus, in order to decide whether an ETV value is low, medium, or high, one needs to create similar plots for different values of *n* and *k*.

### 5.7. Real-World Experiments

Let us study two real-world applications. In the first experiment, we studied English word frequencies. The English word frequency list describes the frequency at which each word appears in a language, based on hundreds of millions of words, collected from open-source subtitles (www.opensubtitles.org, accessed on 14 April 2024) or based on different dictionaries and glossaries (http://en.wiktionary.org/wiki/Wiktionary:Frequency_lists, accessed on 14 April 2024). This results in an alphabet size of approximately 500K words. Our goal was to estimate the entropy of the English word frequency list based on a sample of *n* independent observations from it. Next, we studied the Dow Jones Index (DJI), which demonstrates a time series setting. The DJI dataset contains the daily closing prices of 30 large companies on the U.S. stock exchange [18]. Here, our goal was to estimate the marginal (stationary) entropy of the DJI. For this purpose, we focused on a relatively stationary time period between the years 1990 and 1997 (see Section 5.E. of [18]). The DJI closing values on each day were taken, and the frequency for each value was calculated. The resulting distribution consists of approximately 1600 values, where each symbol represents a unique closing value. Notice that, despite its ordinal nature, we treated each symbol as categorical for the purpose of this experiment.

For each of the datasets above, we drew *n* samples with replacement. The “true” entropy was evaluated from the empirical distribution of the entire dataset, and this entropy was compared to the estimated entropy derived from the samples. This procedure was repeated one hundred times, and the RMSE was calculated for each estimator and sample size. We note that, in both experiments, the underlying distributions (based on the entire datasets) are within the mid-uniform range.

**Figure 11:**
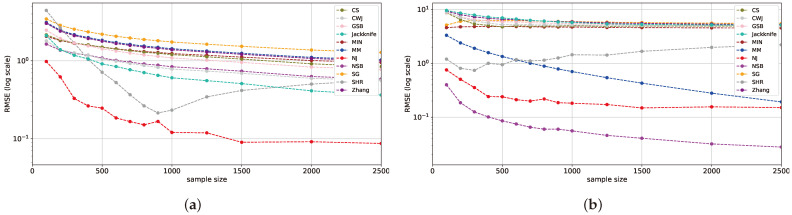
The mean RMSE of selected estimators for the English word and DJI datasets. The English word dataset is presented in (**a**), while the DJI dataset is in (**b**).

We evaluate our conclusions from Section 5.1 as we focus on entropy estimators that are representative of each distribution range. For the English word dataset, Figure 11a shows that NJ, SHR, jackknife, and NSB attain the best results. These findings align with the conclusions in Table 1. The DJI dataset results presented in Figure 11b indicate that NSB, NJ, and SHR have the most competitive performance, further reinforcing previous conclusions.

Overall, the analysis of the real-world datasets aligns with our previous findings in Section 5.1, showing slight differences in the performance of the estimators but maintaining the same general trend of RMSE improvement with the increase in the sample size. In both datasets, NJ, SHR, and NSB provide competitive results, consistent with the top performers in the mid-uniform range.

## 6. Discussion

This research compares twenty-one entropy estimators, including novel neural network-based and polynomial approximate entropy estimators. It focuses on the large-alphabet regime across a variety of distributions from uniform to deterministic, extending the comparative study of [17]. In the analysis, we distinguished between three different distribution ranges, namely, near-, mid-, and far-uniform. Further, we studied the low-, mid-, and high-ETV distances. Our findings indicate that the NJ, NSB, and SHR estimators yield the most favorable results in the near-uniform and low-ETV ranges, as evidenced by the SHR estimator, which also aligns with earlier studies, including [9,17].

From the findings in the near-uniform range, the Bayesian estimator’s performance is highly reliant on the prior, suggesting a future research direction for optimizing the prior of more traditional Bayesian estimators such as SG and MIN to suit the specific problem settings. The NJ, NSB, *unseen*, and CWJ estimators stand out in the mid-uniform range, with NJ outperforming the other three. These findings are consistent with the mid-ETV range.

However, as the distribution shifts toward the far-uniform range, NJ, a neural network-based estimator, begins to under-perform and overgeneralize. Future research could explore this challenge of the NJ estimator, addressing its high bias and lack of convergence as the distribution becomes more deterministic. Interestingly, increasing the network size does not significantly impact the estimator’s performance, indicating that improvements need to be pursued through alternative strategies.

In the far-uniform as well as in the high-ETV range, classical estimators tend to converge, and the top performers include PYM, CDM, and NSB. Notably, NSB yields better outcomes in the high range and mid-range. Future studies could delve further into this estimator, conducting a more detailed comparison to outline the advantages and disadvantages of this estimator in the large-alphabet regime.

The bias analysis reveals that the *polynomial* estimator exhibits the largest bias, presenting another potential direction for research to investigate the intrinsic bias of this estimator. This estimator is designed for the large-alphabet regime, yet improper polynomial approximation results in significant under-performance.

Tying it all together, given an unknown distribution, a generalized assessment of the estimator choice can be performed based on the distribution range. In the low-ETV regime, estimators with a strong generalization ability are likely to produce the best results. These estimators exhibit low bias by correcting it according to the distributions. For mid-ETV values, the most favorable estimators can generalize and adapt to different types of distributions. They are not based on the *plug-in* estimator but instead present an alternative computation method to entropy estimation. Such methods include those based on neural network or linear programming. For a high ETV, estimators based on the *plug-in* method tend to converge and deliver the best performance. This range is characterized by a high ratio of the sample to alphabet size.

## Figures and Tables

**Table 1:** Best entropy estimators for each range, ordered by performance.

Distribution Range	Estimators
Near-uniform	SHR NJ NSB
Mid-uniform	NJ NSB *unseen* *jackknife* BUB SHR
Near-deterministic (far-uniform)	Zhang PYM MM SG SHR NSB GSB *unseen* *jackknife* CWJ *plug-in* CS CDM

## Data Availability

The data presented in this study are available on request from the corresponding author.

## Appendix A. Entropy Estimator Definitions

**Table A1:** Entropy estimator descriptions.

Estimator	Notation	Description
Maximum likelihood [20]	*plug-in*	${\hat{H}}^{ML}=-\sum_{x\in X}{\hat{p}}_{x}log_{2}{\hat{p}}_{x}$.
Miller–Madow correction [15]	MM	${\hat{H}}^{MM}={\hat{H}}^{ML}+\frac{m-1}{2n}$, where *n* is the sample size, $y_{x}=\sum_{i=1}^{n}1\left(X_{i}=x\right)$ is the observed frequency of *x*, and *m* is the number of x∈X such that $y_{x}>0$.
Jackknife [16]	*jackknife*	${\hat{H}}^{JN}=n{\hat{H}}^{ML}-\frac{n-1}{n}{\hat{H}}_{-i}^{ML}$, where ${\hat{H}}_{-i}^{ML}$ is the entropy of the sample, excluding the *i*-th symbol.
Best Upper Bound [2]	BUB	${\hat{H}}^{BUB}=-\sum_{i=0}^{n}a_{i}h_{i}$, where $h_{i}=\sum_{x=1}^{k}1_{\left[y_{x}=i\right]}$, and $a_{i}$ is calculated using an algorithm proposed in [2] that minimizes a “regularized least squares” problem.
Grassberger [10]	GSB	${\hat{H}}^{GSB}=log_{2}n-\frac{1}{n}\sum_{x=1}^{k}y_{x}\left(\psi \left(y_{x}\right)+{\left(-1\right)}^{y_{x}}\int_{0}^{1}\frac{t^{y_{x}-1}}{t+1}dt\right)$, where ψ(.) is the digamma function.
Schürmann [21]	SHU	${\hat{H}}^{SHU}=\psi \left(n\right)-\frac{1}{n}\sum_{x=1}^{k}y_{x}\left(\psi \left(y_{x}\right)+{\left(-1\right)}^{y_{x}}\int_{0}^{\frac{1}{\zeta }-1}\frac{t^{y_{x}-1}}{t+1}dt\right)$.
Chao–Shen * [22,23]	CS	${\hat{H}}^{CS}=-\sum_{x\in X}\frac{{\hat{p}}_{x}^{CS}log_{2}{\hat{p}}_{x}^{CS}}{1-{\left(1-{\hat{p}}_{x}^{CS}\right)}^{n}}$, where ${\hat{p}}_{x}^{CS}=\left(1-\frac{m}{n}\right){\hat{p}}_{x}$.
James–Stein [9]	SHR	${\hat{H}}^{SHR}=-\sum_{x\in X}{\hat{p}}_{x}^{SHR}log_{2}{\hat{p}}_{x}^{SHR}$, where ${\hat{p}}_{x}^{SHR}=\hat{\lambda }t_{x}+\left(1-\hat{\lambda }\right){\hat{p}}_{x}$, with $\hat{\lambda }=\frac{1-\sum_{x=1}^{k}{\left({\hat{p}}_{x}\right)}^{2}}{\left(n-1\right)\sum_{x=1}^{k}{\left(t_{x}-{\hat{p}}_{x}\right)}^{2}}$ and $t_{x}=1/k$.
Bonachela [25]	BN	${\hat{H}}^{BN}=\frac{1}{n+2}\sum_{x=1}^{k}\left[\left(y_{x}+1\right)\sum_{j=y_{x}+2}^{n+2}\frac{1}{j}\right]$.
Zhang [12]	Zhang	${\hat{H}}^{Zhang}=\sum_{\upsilon =1}^{n-1}\frac{1}{\upsilon }Z_{\upsilon }$, where $Z_{\upsilon }=\frac{n^{\upsilon +1}\left[n-\left(\upsilon +1\right)\right]!}{n!}\sum_{x\in X}\left[{\hat{p}}_{x}\Pi_{i=0}^{\upsilon -1}1-{\hat{p}}_{x}-\frac{i}{n}\right]$.
Chao–Wang–Jost [14]	CWJ	${\hat{H}}^{CWJ}=\sum_{1\leq y_{x}\leq n-1}\frac{y_{x}}{n}\left(\sum_{k=y_{x}}^{n-1}\frac{1}{k}\right)+\frac{f_{1}}{n}{\left(1-A\right)}^{-n+1}\left\{-log_{2}A-\sum_{r=1}^{n-1}\frac{1}{r}{\left(1-A\right)}^{r}\right\}$, with $A=\left\{\begin{array}{cc} \frac{2f_{2}}{\left[\left(n-1\right)f_{1}+2f_{2}\right]} & \mathrm{if}\;f_{2}>0\\ \frac{2}{\left[\left(n-1\right)\left(f_{1}-1\right)+2\right]} & \mathrm{if}\;f_{2}=0,f_{1}\neq 0\\ 1 & \mathrm{if}\;f_{2}=f_{1}=0, \end{array}\right.$ where $f_{1}$ is the number of singletons, and $f_{2}$ is the number of doubletons in the sample.
Schürmann–Grassberger [32]	SG	${\hat{H}}^{Bayes}=-\sum_{x\in X}{\hat{p}}_{x}^{Bayes}log_{2}{\hat{p}}_{x}^{Bayes}$, where ${\hat{p}}_{x}^{Bayes}=\frac{y_{x}+1/k}{n+1}$.
Minimax prior [33]	MIN	${\hat{H}}^{Bayes}$ with $a_{x}=\sqrt{n}/k$ and $A=\sqrt{n}$.
Jeffrey [29]	JEF	${\hat{H}}^{Bayes}$ with $a_{x}=1/2$ and A=k/2.
Laplace [31]	LAP	${\hat{H}}^{Bayes}$ with $a_{x}=1$ and A=k.
NSB [11]	NSB	${\hat{H}}^{NSB}=\frac{\int p\left(\zeta ,n\right)H_{\beta }^{m}\left(n\right)d\zeta }{\int p(\zeta ,n)d\zeta }$, where $p\left(\zeta ,n\right)=\frac{\Gamma [k\beta (\zeta )]}{\Gamma [n+k\beta (\zeta )]}\Pi_{x\in X}\frac{\Gamma [y_{x}+\beta \left(\zeta \right)]}{\Gamma [\beta (\zeta )]}$, with $\zeta =\psi_{0}\left(k\beta +1\right)-\psi_{0}\left(\beta +1\right).\psi_{m}\left(x\right)={\left(d/dx\right)}^{m+1}log_{2}\Gamma \left(x\right)$, and $H_{\beta }^{m}\left(n\right)$ is the expectation value of the m-th entropy moment for fixed β. The explicit expression for m=1,2 is in [50].
CDM [34]	CDM	${\hat{H}}^{CDM}=\psi_{0}\left(N+a+1\right)-\sum_{x=1}^{k}\frac{y_{x}+a{\hat{\mu }}_{x}}{N+a}\left(y_{x}+{\hat{\mu }}_{x}+1\right)$, where ${\hat{\mu }}_{x}={\hat{p}}_{x}$.
PYM [35]	PYM	${\hat{H}}^{PYM}=\int E\left[H|x,d,\alpha \right]\frac{p(x|d,\alpha )p(d,\alpha )}{p(x)}d\left(d,\alpha \right)$, where E[H|x,d,α] is the expected posterior entropy for a given (d,α), which determines the prior, and $p\left(x|d,\alpha \right)=\frac{\left(\Pi_{l=1}^{k-1}\left(\alpha +ld\right)\right)\left(\Pi_{i=1}^{k}\Gamma \left(y_{i}-d\right)\right)\Gamma \left(1+\alpha \right)}{\Gamma {\left(1-d\right)}^{k}\Gamma \left(\alpha +n\right)}$.
*polynomial* [19]	*polynomial*	${\hat{H}}^{Polynomial}=\sum_{i=1}^{k}\left(g_{L}\left(y_{i}\right)1_{\left[y_{i}^{\prime }\leq c_{2}log_{2}k\right]}+\left(\phi \left(\frac{y_{i}}{n}\right)+\frac{1}{2n}\right)1_{\left[y_{i}^{\prime }>c_{2}log_{2}k\right]}\right)$, where $g_{L}\left(y_{i}\right)=\frac{1}{n}\left(\sum_{m=0}^{L}\frac{a_{m}}{{\left(c_{1}log_{2}k\right)}^{m-1}}{\left(y_{i}\right)}_{m}+\left(log_{2}\frac{n}{c_{1}log_{2}k}\right)y_{i}\right)$, where *L* is the polynomial degree, $L=\left\lfloor c_{0}log_{2}k\right\rfloor$, $a_{m}$ is an approximate polynomial coefficient, $\phi \left(x\right)=-xlog_{2}x$, $y_{i},y_{i}^{\prime }$ denotes the observed frequencies of the equally split sample, and $c_{0},c_{1},c_{2}>0$ are constants specified in [19].
*unseen* [13]	*unseen*	Algorithmic calculation based on linear programming.
Neural joint entropy estimator [18]	NJ	Neural network estimator based on minimizing the cross-entropy loss.

* Also known as the coverage-adjusted estimator (CAE) [23].

## References

[1] Cover T., Thomas J. (2012). Elements of Information Theory.

[2] Paninski L. (2003). Estimation of Entropy and Mutual Information. Neural Comput..

[3] Antos A., Kontoyiannis I. (2001). Convergence properties of functional estimates for discrete distributions. Random Struct. Algorithms.

[4] Sechidis K., Azzimonti L., Pocock A., Corani G., Weatherall J., Brown G. (2019). Efficient feature selection using shrinkage estimators. Mach. Learn..

[5] Capó E.J.M., Cuellar O.J., Pérez C.M.L., Gómez G.S. Evaluation of input—Output statistical dependence PRNGs by SAC. Proceedings of the IEEE 2016 International Conference on Software Process Improvement (CIMPS).

[6] Madarro-Capó E.J., Legón-Pérez C.M., Rojas O., Sosa-Gómez G., Socorro-Llanes R. (2020). Bit independence criterion extended to stream ciphers. Appl. Sci..

[7] Li L., Titov I., Sporleder C. (2014). Improved Estimation of Entropy for Evaluation of Word Sense Induction. Comput. Linguist..

[8] YAVUZ Z.K., Aydin N., ALTAY G. (2016). Comprehensive review of association estimators for the inference of gene networks. Turk. J. Electr. Eng. Comput. Sci..

[9] Hausser J., Strimmer K. (2009). Entropy inference and the James-Stein estimator, with application to nonlinear gene association networks. J. Mach. Learn. Res..

[10] Grassberger P. (2003). Entropy estimates from insufficient samplings. arXiv.

[11] Nemenman I., Shafee F., Bialek W. Entropy and inference, revisited. Proceedings of the Advances in Neural Information Processing Systems.

[12] Zhang Z. (2012). Entropy estimation in Turing’s perspective. Neural Comput..

[13] Valiant P., Valiant G., Burges C., Bottou L., Welling M., Ghahramani Z., Weinberger K. (2013). Estimating the Unseen: Improved Estimators for Entropy and other Properties. Proceedings of the Advances in Neural Information Processing Systems.

[14] Chao A., Wang Y., Jost L. (2013). Entropy and the species accumulation curve: A novel entropy estimator via discovery rates of new species. Methods Ecol. Evol..

[15] Miller G. (1955). Note on the bias of information estimates. Information Theory in Psychology: Problems and Methods.

[16] Burnham K.P., Overton W.S. (1978). Estimation of the size of a closed population when capture probabilities vary among animals. Biometrika.

[17] Contreras Rodríguez L., Madarro-Capó E.J., Legón-Pérez C.M., Rojas O., Sosa-Gómez G. (2021). Selecting an effective entropy estimator for short sequences of bits and bytes with maximum entropy. Entropy.

[18] Shalev Y., Painsky A., Ben-Gal I. (2022). Neural joint entropy estimation. IEEE Trans. Neural Netw. Learn. Syst..

[19] Wu Y., Yang P. (2016). Minimax Rates of Entropy Estimation on Large Alphabets via Best Polynomial Approximation. IEEE Trans. Inf. Theory.

[20] Strong S.P., Koberle R., Van Steveninck R.R.D.R., Bialek W. (1998). Entropy and information in neural spike trains. Phys. Rev. Lett..

[21] Schürmann T. (2004). Bias analysis in entropy estimation. J. Phys. A Math. Gen..

[22] Chao A., Shen T.J. (2003). Nonparametric estimation of Shannon’s index of diversity when there are unseen species in sample. Environ. Ecol. Stat..

[23] Vu V.Q., Yu B., Kass R.E. (2007). Coverage-adjusted entropy estimation. Stat. Med..

[24] Horvitz D.G., Thompson D.J. (1952). A generalization of sampling without replacement from a finite universe. J. Am. Stat. Assoc..

[25] Bonachela J.A., Hinrichsen H., Munoz M.A. (2008). Entropy estimates of small data sets. J. Phys. A Math. Theor..

[26] Good I.J. (1953). The population frequencies of species and the estimation of population parameters. Biometrika.

[27] Painsky A. (2022). Convergence guarantees for the Good-Turing estimator. J. Mach. Learn. Res..

[28] Zhang Z., Grabchak M. (2013). Bias adjustment for a nonparametric entropy estimator. Entropy.

[29] Krichevsky R., Trofimov V. (1981). The performance of universal encoding. IEEE Trans. Inf. Theory.

[30] Clarke B.S., Barron A.R. (1994). Jeffreys’ prior is asymptotically least favorable under entropy risk. J. Stat. Plan. Inference.

[31] Holste D., Grosse I., Herzel H. (1998). Bayes’ estimators of generalized entropies. J. Phys. Math. Gen..

[32] Schürmann T., Grassberger P. (1996). Entropy estimation of symbol sequences. Chaos: Interdiscip. J. Nonlinear Sci..

[33] Trybula S. (1958). Some problems of simultaneous minimax estimation. Ann. Math. Stat..

[34] Archer E.W., Park I.M., Pillow J.W. Bayesian entropy estimation for binary spike train data using parametric prior knowledge. Proceedings of the Advances in Neural Information Processing Systems.

[35] Archer E., Park I.M., Pillow J.W. (2014). Bayesian Entropy Estimation for Countable Discrete Distributions. J. Mach. Learn. Res..

[36] Kozachenko L.F., Leonenko N.N. (1987). Sample estimate of the entropy of a random vector. Probl. Peredachi Informatsii.

[37] Margolin A.A., Nemenman I., Basso K., Wiggins C., Stolovitzky G., Favera R.D., Califano A. (2006). ARACNE: An algorithm for the reconstruction of gene regulatory networks in a mammalian cellular context. BMC Bioinform..

[38] Daub C.O., Steuer R., Selbig J., Kloska S. (2004). Estimating mutual information using B-spline functions–an improved similarity measure for analysing gene expression data. BMC Bioinform..

[39] Hulle M.M.V. (2005). Edgeworth approximation of multivariate differential entropy. Neural Comput..

[40] Belghazi M.I., Baratin A., Rajeswar S., Ozair S., Bengio Y., Courville A., Hjelm R.D. (2018). Mine: Mutual information neural estimation. arXiv.

[41] Poole B., Ozair S., Van Den Oord A., Alemi A., Tucker G. (2019). On variational bounds of mutual information. Proceedings of the International Conference on Machine Learning.

[42] Song J., Ermon S. (2019). Understanding the limitations of variational mutual information estimators. arXiv.

[43] Hausser J., Strimmer K. (2021). Entropy: Estimation of Entropy, Mutual Information and Related Quantities.

[44] Cao L., Grabchak M. (2015). EntropyEstimation: Estimation of Entropy and Related Quantities.

[45] Eric Marcon B.H. (2023). Entropart: Entropy Partitioning to Measure Diversity.

[46] Archer E., Park M., Pillow J.W. (2015). GitHub—Pillowlab/CDMentropy: Centered Dirichlet Mixture Entropy Estimator for Binary Data.

[47] Archer E., Park M., Pillow J.W. (2020). GitHub—Pillowlab/PYMentropy: Discrete Entropy Estimator Using the Pitman-Yor Mixture (PYM) Prior.

[48] Shalev Y. (2023). GitHub—YuvalShalev/NJEE: Neural Joint Entropy Estimiator, Based on Corss-Entropy Loss.

[49] Archer E., Park M., Pillow J.W. (2021). GitHub—Simomarsili/ndd: Bayesian Entropy Estimation in Python—Via the Nemenman-Schafee-Bialek Algorithm.

[50] Wolpert D.H., Wolf D.R. (1995). Estimating functions of probability distributions from a finite set of samples. Phys. Rev. E.

